# Investigation of the Catalytic Properties of Aluminum Oxide (Al_2_O_3_) and Pyrite (FeS_2_) Using Thermodynamic and Kinetic Parameters

**DOI:** 10.3390/molecules30010142

**Published:** 2025-01-01

**Authors:** Aigul T. Ordabaeva, Zainulla M. Muldakhmetov, Mazhit G. Meiramov, Sergey V. Kim, Shuga B. Kasenova, Serik D. Fazylov

**Affiliations:** 1 Institute of Organic Synthesis and Chemistry of Coal of Kazakhstan Republic, Alikhanov Str., 1, Karaganda 100012, Kazakhstan; iosu.rk@mail.ru (Z.M.M.); vanquishv8@mail.ru (S.V.K.); iosu8990@mail.ru (S.D.F.); 2 Laboratory of Thermochemical Processes, Zh. Abishev Chemical-Metallurgical Institute, Karaganda 100009, Kazakhstan; kasenovashuga@mail.ru

**Keywords:** aluminum oxide, hydrogenation, anthracene, catalyst, kinetics

## Abstract

The kinetics of anthracene hydrogenation was studied using the method of equilibrium kinetic analysis. To determine the diffusion–kinetic characteristics, anthracene hydrogenation was performed at different temperatures (648 K, 673 K, 698 K), at a hydrogen pressure of 3 MPa in the presence of a mixture of pyrite (FeS_2_) and aluminum oxide (Al_2_O_3_) taken at a ratio of 1:1. Chromatographic analysis of anthracene hydrogenation products showed the presence of 9,10-dihydroanthracene (DHA), 1,2,3,4-tetrahydroanthracene (THA), methylnaphthalene (MN), naphthalene (H) and other unidentified compounds. In order to preserve the material balance, a total hydrogenation reaction of anthracene, up to 9,10-dihydroanthracene, was proposed as characterized by the highest rate in the presence of pyrite-based catalysts and aluminum oxide. Calculations of the degrees of rotation of anthracene, reaction constants, and Gibbs energy have shown that with increasing temperature, the reaction becomes more thermodynamically advantageous. Based on the obtained data, Arrhenius dependences were constructed, which made it possible to calculate the activation energies of direct (39.4 kJ/mol) and reverse (13.04 kJ/mol) reactions. Thus, based on the calculations performed, it was found that the process of anthracene hydrogenation in the presence of a mixture of pyrite and aluminum oxide proceeds mainly in the diffusion region.

## 1. Introduction

The determination of the equal and kinetic parameters of a chemical process plays an important role in understanding the nature of the effects of conditions (concentration, temperature, theft, etc.) on the mechanism, speed and composition of the system. The system means a reaction mixture consisting of reagents, intermediates and reaction products [1]. The calculation of equilibrium and kinetic parameters is of great scientific and practical importance, the speed makes it possible to determine the optimal conditions under which the maximum efficiency of various processes is achieved and provides the possibility of forecasting.

Modeling allows us to predict how changes in parameters such as ionic strength, pH, flow rate, and cation exchange impact the retention and release of colloids in porous media. Specifically, modeling based on the relationship between the amount of released colloids and changes in the fraction of the solid surface involved in colloid retention demonstrated that reducing the solution’s ionic strength to 5 mM decreases the number of retained colloids to below 50% of the initial amount [2].

The calculation of equilibrium and kinetic parameters enables the simulation of hydrate dissociation in a porous medium. For example, using the calculated values of the molar dissociation rate constant (36,000 mol Pa^−1^s^−1^m^−2^) and activation energy (77.33–81.08 kJ/mol) allowed determination of the methane release rate from hydrates. Additionally, calculating the medium’s porosity (0.182) made it possible to simulate methane transport through the porous medium, predict gas movement pathways, and understand its interactions with water and hydrate within the porous structure [3].

The use of such modeling methods in the processes of catalytic hydrotreatment of hydrocarbon raw materials makes it possible to better understand the mechanisms underlying the transformation of hydrocarbons and identify key factors affecting the yield of products and the activity of catalysts. For example, modeling of hydroprocessing processes of hydrocarbon raw materials makes it possible to more accurately describe the kinetics of the reaction, taking into account the separate adsorption of hydrogen and hydrocarbons at various active centers of the catalyst, ensuring good agreement with experimental data in a wide range of conditions [4].

Since anthracene is close in its structural properties to polyaromatic hydrocarbons (PAHs), which are part of coal tar and heavy oil residues, it is therefore widely used as a model compound in scientific research on the catalytic hydrotreatment of hydrocarbon raw materials [5].

For the preparation of catalysts, a wide variety of materials are used to achieve high conversion values. For example, it was found that the hydrogenation of anthracene in the presence of nickel deposited on H-β-zeolite in an atmosphere of supercritical carbon dioxide (sc-CO_2_) at a temperature of 100 °C and a pressure of 6.9 MPa leads to a 100% conversion of anthracene [6]. The calculation using the first-order kinetic model in this work showed that the hydrogenation rate constant increases with increasing temperature.

The catalyst obtained by impregnation of activated carbon with isomeric trimethylsilyl trifluoromethanesulphonate (TMSTFMS) and antimony pentachloride (PCA) using microwave irradiation makes it possible to achieve an anthracene conversion of 82.7% 1 h after the start of hydrogenation at an initial hydrogen pressure of 5 MPa and a temperature of 300 °C [7]. Evaluation of the kinetics of the anthracene hydrogenation reaction by superdelocalization (Sr) values for the 9th and 10th positions of carbon atoms in the anthracene molecule showed that mainly 9,10-dihydroanthracene (9,10-DHA) is formed in the hydrogenation products.

Aluminum oxide is used as a substrate for applying active phases (metals and their oxides) for their application in the processes of oxidation, methane reforming, selective catalytic reduction, cracking and hydrotreating in oil refining, hydrogenation and dehydrogenation of various organic compounds [8,9]. These catalysts demonstrate high thermal and chemical stability and remain active after several cycles of use and regeneration [10], which increases their efficiency. 

These properties make it possible to obtain catalysts that provide high conversion values in the hydrogenation of polycyclic compounds. For example, Pd5%/Al_2_O_3_ obtained by incipient wetness impregnation showed an almost complete conversion of naphthalene to decalin with a yield of 99.5% at a temperature of 250 °C and a pressure of 40 bar, demonstrating high values of hydrogenation rate constants [11]. When hydrogenating an even more stable phenanthrene compound in the presence of the CoMo/Al_2_O_3_ catalyst at a temperature of 280 °C and a pressure of 4 MPa, the yield of the target octahydrophenanthrene products (1,8- and 1,10-octahydrophenanthrene) was about 70% of the total reaction products at a relatively high hydrogenation rate constant [12].

Pyrite, both natural and synthesized, exhibits high catalytic activity in a number of chemical processes. Pyrite actively interacts with hydrogen in the process of direct liquefaction of coal, contributing to the rupture of carbon–carbon bonds in coal and the conversion of coal into liquid hydrocarbons [13]. Pyrite exhibits high catalytic activity, contributing to the transformation of heavy fractions such as asphaltenes and pre-asphaltenes into gas and oil (G&O) during the catalytic hydraulic liquefaction of brown coal [14].

Earlier, the authors conducted studies on the catalytic hydrogenation of anthracene using bimetallic zeolite catalysts containing oxide forms of Fe and Co, which, in terms of the yield of 9,10-dihydroanthracene, were close to those using noble metals deposited on aluminum oxide [15].

In this work, the kinetics of anthracene hydrogenation in the presence of a mixture of pyrite and aluminum oxide in a ratio of 1:1, at a hydrogen pressure of 3 MPa and temperatures of 648 K, 673 K, 698 K, were studied by the method of equilibrium-kinetic analysis (EKA) [16]. It was found that an increase in the temperature of the hydrogenation reaction leads to an increase in the fractional conversion of anthracene, equilibrium constants and a decrease in Gibbs energy values.

## 2. Results and Discussion

### 2.1. Derivation of the Differential Model of the Fractional Conversion by the Method of Equilibrium Kinetic Analysis

The values of the fractional conversion of anthracene obtained by hydrogenation of anthracene in the presence of a mixture of pyrite and aluminum oxide at a ratio of 1:1 at different temperatures are shown in Table 1.

From the results presented in Table 1, it can be seen that the fractional conversion of anthracene increases over time with increasing temperature.

The results of calculating the equilibrium fractional conversion at different temperatures are shown in Table 2.

The equilibrium fractional conversion characterizes the fractional conversion of anthracene when a state of equilibrium is reached, when the rates of direct and reverse reactions are equal. These values depend on temperature, but not on time, as they represent the maximum possible fractional conversion under equilibrium conditions. The equilibrium fractional conversion of reaction is used to calculate important thermodynamic quantities such as the equilibrium constant (*K_eq_*) and the Gibbs energy (∆*G*), which allows us to evaluate the thermodynamic benefits of the reaction at different temperatures.

#### 2.1.1. Calculation of the Equilibrium Constant

The obtained equilibrium constants of anthracene hydrogenation at different temperatures are presented in Table 3.

The equilibrium constant *K_eq,T_* is the ratio of the rate of the direct hydrogenation reaction *K_eq,T_* of anthracene to the rate of the reverse reaction (dehydrogenation) at different temperatures. Table 3 shows that with increasing temperature, an increase in *K_eq,T_* is observed, which indicates that under these conditions the reaction tends to go in a forward direction.

The recalculated data on the equilibrium constants *K_eq_* and the calculation results for ${∆G}_{T}^{0}$ are shown in Table 4.

Positive values of the Gibbs energy indicate a low probability of the reaction occurring at a standard pressure of *P* = 1 atm, which is actually observed. As can be seen from Table 4, the Gibbs energy value decreases with increasing hydrogenation temperature.

**Figure 1:**
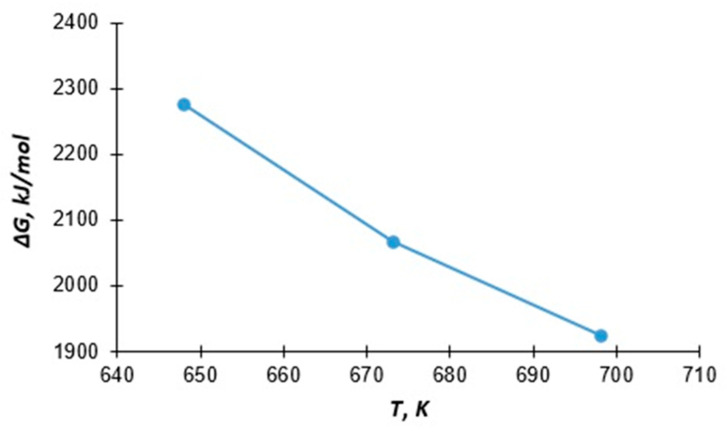
Gibbs energy dependence on temperature.

The dependence of the Gibbs energy on the temperature ${∆G}_{T}^{0}-T$ is shown in Figure 1.

The values of enthalpy ${∆H}_{T}^{0}$ and entropy ${∆S}_{T}^{0}$ of the reaction calculated by the Gibbs-Helmholtz equation were

$${∆H}_{T}^{0}=67.5\frac{kJ}{mol},\;\;{∆S}_{T}^{0}=61.2\;J/(mol\cdot K)$$

Positive values of the enthalpy of the reaction indicate an endothermic process, i.e., proceeding with heat absorption, which is consistent with its more complete passage with increasing temperature [17]. Positive values of the entropy of the reaction indicate an increase in the disorder of the system, which may be due to an increase in the number of saturated bonds as a result of partial hydrogenation of anthracene to 9.10-dihydroanthracene. During the hydrogenation of anthracene, one of its aromatic rings loses part of its aromaticity, which can lead to a change in the structural ordering of the molecule [18]. As a result, there is an increase in the variety of possible conformational states of the molecule, which, in turn, causes an increase in the entropy of the system.

The results of calculations of the rate constants *K_2_* of the reverse reaction are presented in Table 5.

**Figure 2:**
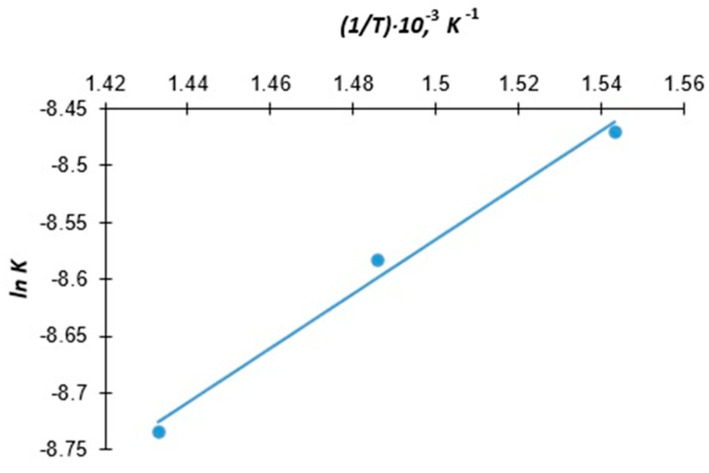
Arrhenius dependence of the velocity constant on the inverse temperature.

The Arrhenius dependence of the velocity constant on the inverse temperature is shown in Figure 2.

#### 2.1.2. Calculation of the Rate Constant of Direct Reaction and Reverse Reactions

The initial data and the results of calculating the rate constants and activation energies of the forward and reverse reactions are shown in Table 6.

As can be seen from Table 6, the rate constants of the direct reaction increase with increasing temperature, and for the reverse reaction, a decrease in the rate constants is observed. The insignificant amount of activation energy is probably caused by the action of a catalyst that facilitates the dissociation of hydrogen molecules into atoms.

Thus, there is reason to believe that the mixture of pyrite and aluminum oxide used in the reaction acts catalytically in both directions of the reaction, which, on the one hand, ensures its implementation in the selected range of temperatures and, on the other hand, explains its moderate total rate.

An increase in the rate constant of the direct reaction of anthracene hydrogenation with an increase in temperature is associated, according to the Arrhenius equation, with an increase in the kinetic energy of molecular motion, which leads to more effective collisions of reagent molecules in active centers on the surface of the catalyst, contributing to an increase in the reaction rate [19]. Thus, an increase in temperature increases the rate of direct reaction due to increased kinetic processes and reduces the likelihood of a reverse reaction due to thermodynamic and catalytic effects that support the formation and stability of hydrogenated products [20]. An increase in the rate of the anthracene hydrogenation reaction with an increase in temperature may indicate that the catalyst promotes effective adsorption of reagents on the active centers of its surface, which reduces the total energy of the system, thermodynamically stabilizes the hydrogenated products formed, and makes them less susceptible to reverse reaction [21]. When products have minimal Gibbs free energy, they are in a more stable state, which makes the reverse reaction thermodynamically less advantageous.

The study presented in this article shows that when anthracene is hydrogenated in the presence of a mixture of Al_2_O_3_ and FeS_2_, the Gibbs energy decreases with increasing temperature, confirming the thermodynamic stability of product formation.

The initial data and the results of calculating the equilibrium fractional conversion of anthracene *α_eq_* (starting from 60 min) are shown in Table 7.

The results of calculating the fractional conversion of anthracene *α_τ_* depending on time are shown in Table 8.

The analysis of the calculations made allows us to conclude that the process of anthracene hydrogenation proceeds mainly in the diffusion region. Obviously, this manifests the action of a catalyst that transfers the process from the chemical stage to the diffusion stage, which can be observed during the hydrogenation of anthracene in the presence of a porous sulfidated catalyst [22]. Since large molecules are more difficult to penetrate into the pores of the catalyst, the reaction proceeds mainly on the outer surface, and the inner part of the catalyst is underutilized due to diffusion limitations. For anthracene, this is manifested in a decrease in the overall efficiency of the hydrogenation reaction due to difficulties with diffusion.

## 3. Materials and Methods

### 3.1. Preparation of Catalysts

Aluminum oxide (Al_2_O_3_) and pyrite (FeS_2_) were calcined in a muffle furnace SNOL 7.2/1100 (Lithuania, European Union). Al_2_O_3_ was calcined at 600 °C and FeS_2_ was calcined at 300 °C for 4 h. After calcination, pyrite and aluminum oxide were mixed with each other in a ratio of 1:1.

### 3.2. Hydrogenation of Anthracene

Anthracene hydrogenation was carried out in a CJF-0.05 reactor manufactured by Zhengzhou Keda Machinery and Instrument (Zhengzhou, China) with a capacity of 0.05 L, equipped with an agitator, temperature and pressure sensors. The initial pressure was 3 MPa. Anthracene hydrogenation was performed at temperatures: 648 K, 673 K, 698 K, at an initial hydrogen pressure of 3 MPa and a heating rate of 10 °C/min in the presence of a mixture (1:1) of pyrite and aluminum oxide. After completion of hydrogenation and cooling of the reactor to room temperature, the resulting reaction mixture was dissolved in benzene and analyzed by gas-liquid chromatography.

The identification of anthracene hydrogenation products was carried out using a Crystalux 4000 M chromatograph with a plasma ionization detector on a Zebron ZB-5 column (Phenomenex, Torrance, CA, USA): column type—capillary; length—30 m; inner diameter—0.32 mm; thickness of the stationary phase—0.50 microns. The temperature range is from 60 to 360 °C. The temperature regime was maintained from 120 to 260 °C at a rate of 12 °C per minute. Quantitative calculation of chromatographic data was carried out using the NetChrom V 2.1 program using the percentage normalization method. Calculation of concentration (mass. %) of hydrogenation products is produced by peak area. The anthracene conversion was calculated based on the data obtained from gas chromatographic analysis of the concentrations of hydrogenation products (wt. %) and the concentration of anthracene remaining after hydrogenation. 

Chromatographic analysis of anthracene (A) hydrogenation products showed the presence of 9,10-dihydroanthracene (DHA), 1,2,3,4-tetrahydroanthracene (THA), methylnaphthalene (MN), naphthalene (N) and other unidentified compounds. Therefore, in order to preserve the material balance, a total hydrogenation reaction of anthracene to 9,10-dihydroanthracene was proposed, as characterized by the highest rate in the presence of a mixture of pyrite and aluminum oxide (1:1):C_14_H_10_ + H_2_

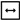
 C_14_H_12_
A + H_2_

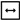
 DHA

Conditions for the hydrogenation of anthracene in the presence of aluminum oxide:

The density of anthracene is *ρ* (A)—1.283 g/cm^3^;

The density of 9,10-dihydroanthracene *ρ* (DHA) is 0.880 g/cm^3^;

The free volume of the autoclave (taking into account the loaded attachment and the cover from the thermocouple) is 50 cm^3^;

The initial pressure of hydrogen is *P*_0_ = 30 atm at an initial temperature of *T*_0_ = 293 K.

The temperature range during hydrogenation is 648–698 K. At these temperatures, A (anthracene) and DHA (9,10-dihydroanthracene) are mutually soluble liquids.

Initial volume of anthracene (*V*_0_):

*V*_0_(A) = *m*(A)/*ρ* (A) = 3/1.283 = 2.338 cm^3^,

where *m*(A) is the weight of the anthracene sample (g), *ρ*(A) is the density of anthracene (g/cm^3^).

When anthracene is completely converted into 9,10-dihydroanthracene, a product with a mass will be obtained:

$$\frac{3(12.011\cdot 14+1.0079\cdot 12)}{\left(12.011\cdot 14+1.0079\cdot 10\right)}=3.216\;g$$

The volume of 9,10-dihydroanthracene *V*(DHA) is found in the same way as for anthracene: *V*(DHA) = *m*(DHA)/*ρ*(DHA) = 3.216/0.880 = 3.654 cm^3^;

Maximum volume change: *V*(DHA) − *V*(A) = 3.654 − 2.338 = 1.316 cm^3^;

In relation to the free volume of the autoclave occupied by hydrogen, this is: 

50 cm^3^—100%

1.316 cm^3^—*X*%,

from where we get: *X* = 1.316 · 100/50 = 2.632%;

Taking into account the accuracy of analytical definitions, such a change in free volume can be ignored.

Initial number of moles of hydrogen (*n_b,o_*):

$$n_{b,o}=\frac{P_{o\cdot }V}{RT}=\frac{30\cdot 10^{6}\cdot 50\cdot 10^{-6}}{10.2\cdot 8.31441\cdot 293}=6.034\cdot 10^{-2}\mathrm{mol}$$

where *P_o_* is pressure, Pa; *V* is volume, m^3^; is the universal gas constant, J/(mol·K); *T* is the temperature, K.

The values of the fractional degrees of transformation of anthracene obtained by hydrogenation of anthracene in the presence of a mixture of pyrite and aluminum oxide at different temperatures are shown in Table 1.

### 3.3. Derivation of a Differential Model of the Degree of Transformation by the Method of Equilibrium Kinetic Analysis

The task of the first stage of equilibrium kinetic analysis is to derive the equation of the total reaction rate, taking into account the retarding effect of the reverse reaction rate, while reducing the total number of variables to only two, in this case to the degree of transformation of *α* and *τ*, in order to further separate the variables and calculate integrals to directly express the dependence of *α* on *τ* and find the equilibrium characteristic, in in this case, *α_eq_* and *τ*, as well as the rate constants of the forward and reverse reactions and the equilibrium constants, this allows the processing of these characteristics, depending on temperature, to determine the activation energy, thermal effect and other thermodynamic functions of the reaction. The strictest expression of the law of action of masses and the equilibrium constant is realized through the activity of reagents.

For a direct reaction, the speed is as follows:

(1) $$\upsilon_{1}=k_{1}a_{A}a_{DHA}$$

where *υ*_1_ is the rate of direct reaction; *k*_1_ is the rate constant of direct reaction; *a_A_*, *a_DHA_*—are the activity of anthracene (*A*) and 9,10-dihydroanthracene (*DHA*), respectively;

for the reverse reaction:

(2) $$\upsilon_{2}=k_{2}a_{DHA}$$

where *υ*_2_ is the rate of the reverse reaction; *k*_2_ is the rate constant of the reverse reaction

The total rate of the anthracene hydrogenation reaction is equal to the difference in the rates of the forward and reverse reaction:

(3) $$\upsilon_{1-2}=\upsilon_{1}-\upsilon_{2}=k_{1}a_{A}a_{b}-k_{2}a_{DHA}$$

Determining the rate of forward and reverse reactions provides an understanding of the dynamics of the system under conditions when the reverse reaction slows down the process; therefore, it is necessary to take into account the contribution of both reactions for accurate modeling of the process and determination of rate constants [23]. In addition, the overall rate of the hydrogenation process may be influenced by the processes of deactivation of the catalyst [24] and processes that interfere with mass transfer [25]. Mass transfer restrictions can have a significant impact on the kinetics of the reaction, leading to distortion of process parameters such as activation energy and reaction order. The nature of the catalyst and its structural features can also affect the distribution of active centers and, consequently, the kinetics and selectivity of the process [26,27]. Also, during the hydrogenation of aromatic compounds, the effects of inhibition associated with the processes of competing adsorption of aromatic compounds on the surface of the catalyst are possible. This phenomenon is caused by the fact that organic compounds with two or more aromatic rings have higher adsorption constants compared to aromatic compounds with one ring, and occupying the active centers of the catalyst, they prevent the hydrogenation of other compounds until they themselves are completely hydrogenated [18].

The activity of reagents in the liquid phase can be expressed in terms of their molar fractions:

(4) $$a_{A}={n_{A}/(n}_{A}+n_{DHA})$$

(5) $$a_{DHA}={n_{DHA}/(n}_{A}+n_{DHA})$$

where *n_A_*, *n_DHA_* are the molar fractions of anthracene (*A*) and 9,10-dihydroanthracene (*DHA*), respectively.

The activity of hydrogen gas at moderate pressure can be identified with its pressure:

(6) $$a_{H}=P_{H}$$

Based on the Clapeyron–Mendeleev equation, the pressure of hydrogen can be expressed in terms of the number of moles:

(7) $$P_{H}=n_{H}RT/V$$

where *n_H_* is the molar fraction of hydrogen.

As a result of the experiment, the fractional conversion *α* of anthracene into dihydroanthracene is determined; therefore, for a single expression of all variables in terms of consumption and accumulation of reagents, it is advisable to recalculate their activity by α. For dihydroanthracene, Formula (5) makes sense of the degree of conversion of *A* to *DHA*. Since *nA + nDHA* = *n_A,_*_0_, where *n_A,_*_0_ is the initial mole fraction of anthracene, then:

(8) $$a_{DHA}={n_{DHA}/(n}_{A}+n_{DHA})={n_{DHA}/n}_{A,0}=\alpha$$

For anthracene, due to the fact that *n_A_* = *n_A,_*_0_ − *n_DH_*_A_, we obtain:

(9) $$a_{A}={n_{A}/(n}_{A}+n_{DHA})={n_{A}/n}_{A,0}=\left(n_{A,0}-n_{DHA}\right)/n_{A,0}=n_{A,0}/n_{A,0}-n_{DHA}/n_{A,0}=1-\alpha$$

For hydrogen, according to the stoichiometry of the reaction, the molar loss is equal to the molar fraction *A*:

(10) $$n_{H}=n_{H,o}-\alpha n_{A,0}$$

where *n_H,_*_0_ is the initial molar fraction of hydrogen.

Therefore, expression (50) will take the form:

(11) $$P_{H}=(n_{H,0}-\alpha n_{A,0})RT/V$$

Thus, the reaction rate can be expressed in terms of α by substituting in (3), the Formulas (8), (9), and (11):

(12) $$\upsilon_{1-2}=\frac{d\alpha }{d\tau }=k_{1}(1-\alpha )(n_{H,0}-\alpha n_{A,0})RT/V-k_{2}\alpha$$

Since *K_eq_ = k*_1_*/k*_2_, where *K_eq_* is the equilibrium constant, it makes sense to unify variables to express the equilibrium constant in terms of the equilibrium value of the fractional conversion α*_eq_*. At equilibrium, the rates of the forward and reverse reactions are equal, and the fractional conversion is equal to *α_eq_*. Expressing the velocities *υ*_1_ and *υ*_2_ in Formulas (1) and (2) through *α* by substituting activity Formulas (8), (9) and (11) in them and referring them to equilibrium conditions, we obtain:

(13) $$V_{1eq}=k_{1}\left(1-\alpha_{eq}\right)\left(n_{H,0}-\alpha_{eq}n_{A,0}\right)RT/V_{1}$$

(14) $$V_{2P}=k_{2}\alpha_{eq}$$

From the equality of these speeds:

(15) $$k_{1}(1-\alpha_{eq})(n_{H,0}-\alpha_{eq}n_{A,0})RT/V=k_{2}\alpha_{eq}$$

It was found:

(16) $$\frac{k_{1}}{k_{2}}=\frac{\alpha_{eq}}{(1-\alpha_{eq})(n_{H,o}-\alpha_{eq}n_{A,o})RT/V_{1}}=K_{eq}$$

We substitute this expression in (13) instead of *k*_1_/*k*_2_:

(17) $$\frac{d\alpha }{d\tau }=K_{2}\left[\frac{\alpha_{eq}(1-\alpha )(n_{H,0}-\alpha n_{A,0})RT/V}{(1-\alpha_{eq})(n_{H,0}-\alpha_{p}n_{A,0})RT/V}-\alpha \right]$$

Or after the reduction to (RT/V) we obtain:

(18) $$\frac{d\alpha }{d\tau }=K_{2}\left[\frac{\alpha_{eq}(1-\alpha (n_{H,\;\;0}-\alpha n_{A,0})}{(1-\alpha_{eq})(n_{H,0}-\alpha_{eq}n_{A,0})RT}-\alpha \right]$$

This is the differential kinetic model of the process under study, corresponding to the requirement of the EKA method.

Firstly, it has kinetic (*K*_2_) and equilibrium (*α_eq_*) reaction characteristics. Secondly, there are only two variables in it—*α* and *τ*, which can be separated. Of the constants, *n_H,0_* and *n_A,_*_0_ are known in advance, and the unknown constants for each temperature are *K_2_* and *α_eq_*. Since the experimental data are *α* and *τ*, in order to process them according to a mathematical model, it is necessary to transfer it from a differential form to an integral one, i.e., to release *α* and *τ* from under the sign of the differential.

### 3.4. Obtaining an Integral Model

To simplify the transformation, we introduce a notation for one of the constant parts:

(19) $$\frac{\alpha_{eq}}{\left(1-\alpha_{eq})(n_{H,0}-\alpha_{eq}n_{A,0}\right)}=A$$

In this case, Formula (19) will take the form:

(20) $$\frac{d\alpha }{d\tau }=k_{2}\left[A(1-\alpha )(n_{H,0}-\alpha n_{A,0})-\alpha \right]$$

Next, we will expand the brackets in the first term:

(21) $$\frac{d\alpha }{d\tau }=k_{2}\left[An_{H,0}-An_{H,0}\alpha -An_{A,0}\alpha +An_{A,0}\alpha^{2}-\alpha \right]$$

Then, we group the terms with respect to the variable *α*:

(22) $$\frac{d\alpha }{d\tau }=k_{2}\left[An_{A,0}\alpha^{2}-(An_{H,0}+An_{A,0}+1)+\alpha +An_{H,0}\right]$$

Introduce the notation:

(23) $$An_{A,0}=a$$

(24) $$-(An_{H,0}+An_{A,0}+1)=b$$

(25) $$An_{H,0}=c$$

After substituting into Formula (23) we obtain:

(26) $$\frac{d\alpha }{d\tau }=k_{2}(a\alpha^{2}+b\alpha +c)$$

Separate the variables and introduce them under the signs of integrals:

(27) $$\int_{0}^{\alpha }\frac{d\alpha }{a\alpha^{2}+b\alpha +c}=\int_{0}^{\tau }k_{2}d\tau$$

The right integral refers to an elementary function and is revealed as follows:

(28) $$\int_{0}^{\tau }k_{2}d\tau =k_{2}\int_{0}^{\tau }d\tau =k_{2}{\left.\tau \right|}_{0}^{\tau }=k_{2}(\tau -0)=k_{2}\tau$$

where *C*_1_ and *C*_2_ are the integration constants.

Therefore, first of all, it is necessary to determine the determinant by substituting into it the designations *a*, *b* and *c* from (24)–(26):

(29) $$\Delta =4ac-b^{2}=4A{п}_{A,0}An_{b,0}-{\left[-\left(An_{b,0}+A{п}_{A,0}+1\right)\right]}^{2}==4A^{2}n_{A,0}{п}_{b,0}-{\left[\left(An_{b,0}+A{п}_{A,0}+1\right)\right]}^{2}==\;4A^{2}n_{A,0}{п}_{b,0}-\left(An_{b,0}+A{п}_{A,0}+1\right)\left(An_{b,0}+A{п}_{A,0}+1\right)==4A^{2}n_{A,0}{п}_{b,0}-A^{2}{п}_{b,0}^{2}-A^{2}n_{A,0}{п}_{b,0}-An_{b,0}-\;A^{2}n_{A,0}{п}_{b,0}==-A^{2}n_{A,0}^{2}-An_{A,0}-An_{b,0}-An_{A,0}-1==2A^{2}n_{A}{п}_{b,0}-A^{2}{п}_{b,0}^{2}-2An_{b,0}-A^{2}{п}_{A,0}^{2}-2A{п}_{A,0}-1$$

From the result obtained, we select and transform the fragment:

$$-A^{2}n_{A,0}^{2}-2A^{2}n_{A}{п}_{b,0}-A^{2}{п}_{b,0}^{2}=-A^{2}\left({п}_{A,0}^{2}-2n_{A,0}{п}_{b,0}+{п}_{b,0}^{2}\right)=-A^{2}{\left({п}_{A,0}-{п}_{b,0}\right)}^{2}$$

Returning this fragment to the determinant:

(30) $$\Delta =-2An_{b,0}-2A{n_{A}}_{,0}-1-A^{2}{\left({п}_{A,0}-{п}_{b,0}\right)}^{2}=-\left[2An_{b,0}+2A{n_{A}}_{,0}+1+A^{2}{\left({п}_{A,0}-{п}_{b,0}\right)}^{2}\right]\;<0,$$

since the expression in square brackets is purposeful and generally positive:

*A* > 0, because $n_{b,0}>\alpha_{eq}{n_{A}}_{,0}$—due to the excess of molar hydrogen content relative to anthracene, *a*_2_*α_eq_* < 1 (see Formula (20));

${\left({п}_{A,0}-{п}_{b,0}\right)}^{2}>0$, since after squaring any difference becomes positive.

Thus, the solution of the integral under discussion in this case will be option (31), which, taking into account the lower and upper limits, becomes definite, i.e., without an integration constant:

(31) $$\int_{0}^{\alpha }\frac{d\alpha }{a\alpha^{2}+b\alpha +c}=\frac{1}{\sqrt{-\Delta }}\ln\;\frac{2a\alpha +b-\sqrt{-\Delta }}{2a\alpha +b+\sqrt{-\Delta }}{|}_{0}^{\alpha }=\frac{1}{\sqrt{-\Delta }}\left(\ln\;\frac{2a\alpha +b-\sqrt{-\Delta }}{2a\alpha +b+\sqrt{-\Delta }}-\ln\;\frac{b-\sqrt{-\Delta }}{b+\sqrt{-\Delta }}\right)=\;\frac{1}{\sqrt{-\Delta }}\ln\;\frac{\left(2a\alpha +b-\sqrt{-\Delta }\right)\left(b+\sqrt{-\Delta }\right)}{\left(2a\alpha +b+\sqrt{-\Delta }\right)\left(b-\sqrt{-\Delta }\right)}=\frac{1}{\sqrt{-\Delta }}ln\left[\left(\frac{2a\alpha }{b-\sqrt{-\Delta }}+1\right)/\left(\frac{2a\alpha }{b+\sqrt{-\Delta }}+1\right)\right]$$

Substituting the left and right integrals in (28), we obtain an integral kinetic model of anthracene hydrogenation:

(32) $$\frac{1}{\sqrt{-\Delta }}\ln\;\left[\left(\frac{2a\alpha }{b-\sqrt{-\Delta }}+1\right)/\left(\frac{2a\alpha }{b+\sqrt{-\Delta }}+1\right)\right]=K_{2}\;\tau$$

Determination of the equilibrium fractional conversion of *α_eq_*. In the resulting Equation (35) there are two unknown quantities *K*_2_ and *α_eq_*. Therefore, they cannot be determined from a single experimental point. Two methods have been developed for finding *K*_2_ and *α_eq_* over the entire set of experimental points and over two points with a search of all independent combinations of pairs of points. The second option is simpler, its essence is as follows.

For the *i*-th point, Equation (35) will take the form:

(33) $$\frac{1}{\sqrt{-\Delta }}\ln\;\left[\left(\frac{2a\alpha_{i}}{b-\sqrt{-\Delta }}+1\right)/\left(\frac{2a\alpha_{i}}{b+\sqrt{-\Delta }}+1\right)\right]=K_{2}\;\tau_{i}$$

For the *j*-th point:

(34) $$\frac{1}{\sqrt{-\Delta }}\ln\;\left[\left(\frac{2a\alpha_{j}}{b-\sqrt{-\Delta }}+1\right)/\left(\frac{2a\alpha_{j}}{b+\sqrt{-\Delta }}+1\right)\right]=K_{2}\tau_{j}\;$$

By dividing (36) by (37), we immediately get rid of the unknown value *K_2_*, as well as of the fraction before the logarithm sign:

(35) $$\frac{\ln\;\left[\left(\frac{2a\alpha_{i}}{b-\sqrt{-\Delta }}+1\right)/\left(\frac{2a\alpha_{i}}{b+\sqrt{-\Delta }}+1\right)\right]}{\ln\;\left[\left(\frac{2a\alpha_{j}}{b-\sqrt{-\Delta }}+1\right)/\left(\frac{2a\alpha_{j}}{b+\sqrt{-\Delta }}+1\right)\right]\;}=\frac{\tau_{i}}{\tau_{j}}$$

In addition to the experimental points *α_i_*, *τ_i_* and *α_j_*, *τ_j_*, this equation contains the algebraic quantities *a*, *b* and Δ, which, according to the designations (20), (24)–(26), include the desired degree of reaction *α_eq_*. Direct substitution of the disclosed expressions for a, b, and Δ and Δ *= 4ac* − *b*^2^ is possible in principle, but will make Equation (38) too cumbersome. In any case, the liberation of *α_eq_* from this equation is algebraically impossible; therefore, the finding of *α_eq_* is performed by numerical solution, i.e., by selecting such a value of aeq, in which the left part (38) becomes equal to the right, having a numerical value of *τ_i_*/*τ_j_*.

For this purpose, in order to unify calculations, the right part is set with the condition *τ_i_* < *τ_j_*, which ensures the inequality *τ_i_*/*τ_j_* < 1, facilitating the selection of *α_eq_*. The selection procedures are well developed in well-known software packages, for example, by the method of wine division, but it is quite possible to use a simple procedure for gradually increasing the value of *α_eq_*, starting with tenths, then moving to hundredths and thousandths. In this case, the first search value *α_eq_* can be taken at least *α_j_*, i.e., *α_eq_* ≥ *α_j_*. The transition to more precise digits should occur if the left part (38) becomes larger than the right. In this case, you need to go back a step in the search and start the next digit from one, etc., until the last digit shows an excess of the left part (38) over the right. Here, it should be borne in mind that the number of digits are chosen by one more than is justified by the accuracy of the experimental data. If you want to calculate the *α_eq_* to thousandths, then the search calculations should be carried out to ten thousandths, so that you can then round the result to the nearest thousandths.

It is also necessary to determine the total number of pairs of calculated points. If the number of independent definitions (i.e., under different conditions, in this case durations) is equal to *n*, then the number of non-repeating combinations of *n* by *τ* is equal to:

(36) $$C_{n}^{\tau }=\frac{n!}{\tau !(n-k)!}$$

(37) $$N=C_{n}^{\tau }=\frac{n!}{2!(n-2)!}=\frac{(n-2)!(n-1)!}{2(n-2)!}=\frac{\left(n-1\right)n}{2}$$

The selection is performed for each isotherm separately, and then the average value $\bar{\alpha_{eq}}$ for each isotherm is found.

The program of the *α_eq_* search calculation was carried out on a PC, pre-formulas (38), (20), (24)–(26) brought together:

(38) $$\frac{\ln\;\left[\left(\frac{2a\alpha_{i}}{b-\sqrt{-\Delta }}+1\right)/\left(\frac{2a\alpha_{i}}{b+\sqrt{-\Delta }}+1\right)\right]}{\ln\;\left[\left(\frac{2a\alpha_{j}}{b-\sqrt{-\Delta }}+1\right)/\left(\frac{2a\alpha_{j}}{b+\sqrt{-\Delta }}+1\right)\right]\;}=\frac{\tau_{i}}{\tau_{j}}$$

where:

$$a=An_{A,0},$$

$$C=An_{b,0},$$

$$b=-\left(An_{b,0}+A{n_{A}}_{,0}+1\right)=-\left(c+a+1\right),$$

$$A=\frac{\alpha_{p}}{\left(1-\alpha_{p}\right)\left(n_{b,0}-\alpha_{p}n_{A,0}\right)},$$

$$\Delta =4ac-b^{2}$$

The calculation results are shown in Table 2 (experimental data on *α_eq_* and *τ* are taken from Table 1). Thus, the first task is solved to determine the equal degree of reaction at different temperatures. The equilibrium constants can be calculated from these data.

### 3.5. Calculation of the Equilibrium Constant

Substituting into Formula (17) the found values of *α_eq,T_*, as well as *n_b,_*_0_ = 6.034⋅10^−2^ mol, *n_A,_*_0_ =1.683⋅10^−2^ mol, *R* = 8.31441 J⋅mol^−1^K^−1^, *V* = 5⋅10^−5^ m^3^ and the corresponding temperature values, we find *K_eq,T_*. The obtained equilibrium constants of anthracene hydrogenation at different temperatures are presented in Table 3.

The dimension of the equilibrium constant according to Formula (17) stands for

$$\left\lfloor \frac{m^{3}mol\cdot K}{J\cdot K\cdot mol}=\frac{m^{3}}{J}=\frac{m^{3}\cdot s^{2}}{kg\cdot m^{2}}=\frac{ms^{2}}{kg}={Pa}^{-1}\right\rfloor ,$$

which is consistent with the determining value of the hydrogen pressure.

We performed the calculation of the Gibbs energy of the hydrogenation reaction. The calculation is based on the Van’t–Goff equation:

(39) $${∆G}_{T}^{0}=-RTlnK_{eq}$$

Due to the consideration of the standard state in atm, and not in Pa, the equilibrium constant should be recalculated to the dimension atm^−1^. The conversion factor Pa =9.87⋅10^−6^ atm and, accordingly, Pa^−1^ = 101317 atm^−1^. The recalculated *K_eq_* data and the calculation results for ${∆G}_{T}^{0}$ are shown in Table 4.

The obtained data are placed on a straight line in the coordinates ${∆G}_{T}^{0}-T$ without significant deviation, which allows them to be used to obtain additional thermodynamic information. The dependence of Gibbs energy on temperature is shown in Figure 1.

We performed the calculation of the thermal effect and entropy of the reaction. Due to the rectilinear placement of data in the coordinates ${∆G}_{T}^{0}-T$ (Figure 1), it is possible to process this data using the Gibbs–Helmholtz equation:

(40) $${∆G}_{T}^{0}={∆H}_{T}^{0}-T{∆S}_{T}^{0}$$

In the approximation ${∆H}_{T}^{0}-const$, ${∆S}_{T}^{0}-const$—const in the studied temperature range or for the average temperature range:

(41) $${∆G}_{T}^{0}={∆H}_{673}^{0}-T{∆S}_{673}^{0}$$

With a slight deviation of the described points from the straight line, a graphical definition of the free term and the proportionality coefficient is acceptable, for example, using the two-point method. Such points are taken:

$$T_{1}=648\;K,\;{∆G}_{648}^{0}=2277.2\;J/mol,\;T_{2}=698\;K,\;{∆G}_{698}^{0}=2277.2\;J/mol$$

According to the general form of the equation of a straight line using the two-point method:

(42) $$Y=Y_{1}+\frac{Y_{2}-Y_{1}}{X_{2}-X_{1}}\left(X-X_{1}\right)$$

For our variables, we will find:

$${∆G}_{T}^{0}=2277.2+\frac{\left(1924.2-2277.2\right)}{698-648}\left(T-648\right)=2277.2-61.2\left(T-648\right)=6743-61.2T$$

From comparison with Equation (43), we find:

$${∆H}_{T}^{0}=67.5\;\mathrm{kJ/mol}$$

$${∆S}_{T}^{0}=61.2\;\;\mathrm{J/(mol\cdot K)}$$

Due to the explicit rectilinear placement of data in the coordinates ${∆G}_{T}^{0}-T$, the extrapolation to the standard temperature can be carried out in the Ulich approximation:

(43) $${∆G}_{T}^{0}={∆H}_{298}^{0}-T{∆S}_{298}^{0}$$

In this case, the found values of the enthalpy and entropy of the reaction can be attributed to the standard temperature:

$${∆H}_{298}^{0}=67.5\frac{kJ}{mol},\;\;{∆S}_{298}^{0}=61.2\;\frac{J}{mol\cdot K}$$

These data can be used to evaluate unknown standard characteristics of anthracene and 9,10-dihydroanthracene (DHA).

Next, we proceed to the calculation of the kinetic characteristics of the reaction.

We performed the calculation of the rate constant of the reverse reaction. The found equilibrium degrees of transformation of *α_eq_* allow using the model (35) to calculate the value of *K*_2_ by algebraic transformation (35):

(44) $$\frac{1}{\tau \sqrt{-\Delta }}\ln\;\left[\left(\frac{2a\alpha }{b-\sqrt{-\Delta }}+1\right)/\left(\frac{2a\alpha }{b+\sqrt{-\Delta }}+1\right)\right]$$

Each experimental point in the isotherm should be substituted into this formula, followed by averaging the resulting *K*_2_ value. As a result of the calculations, the data of the rate constants of the reverse reaction were obtained, which are presented in Table 5.

The Arrhenius dependence of the velocity constant (*ln K*_2_) on the inverse temperature (1*/T*) is shown in Figure 2.

### 3.6. Calculation of the Rate Constants of Forward and Reverse Reactions

Since *K_eq_ = K*_1_*/K*_2_, the found values of *K_eq_* and *K*_2_ can be used to find *K_1_* using the formula *K*_1_ *= K_eq_ · K*_2_.

Data on the dependences of *K*_1_ and *K*_2_ on the temperature of the Arrhenius coordinates were used to calculate the activation energy of the forward and reverse reactions. The initial data and the results of calculating the rate constants and activation energies of the forward and reverse reactions are shown in Table 6.

The obtained data make it possible to express analytically with numerical coefficients and the dependence of the degree of response on the duration, calculate and graphically represent this dependence in the full range of duration for each isotherm and for any given degree of approximation to the equilibrium degree of approximation to the equilibrium degree of response.

Based on the low values of the activation energy, it can also be concluded that the process of catalytic hydrogenation of anthracene takes place in boundary conditions between the kinetic and diffusion regions. Therefore, the second diffusion model of the equilibrium kinetic analysis (EKA) method was used.

The diffusion–kinetic model has the following form:

(45) $$\frac{da}{d\tau }=\frac{D}{\delta }F(P_{H_{2\;}}-P_{H_{2,\;\;eq}})\;$$

where is the diffusion coefficient, (m^2^/s); *F* is the surface area of the catalysts, (m^2^/g); *δ* is the thickness of the diffusion layer, (m); $P_{H_{2,\;\;eq}}$ is the equilibrium pressure of hydrogen.

Hydrogen is supplied to the autoclave with an excess against the required reaction.

Using the number of moles of each reaction component,

A + H_2_ = DHA

we obtain the condition $n_{H_{2}>}n_{A}$. This must be checked, and this is respected in this case. Otherwise, the reaction may stop not because it has reached equilibrium, but because there was not enough hydrogen.

Based on this, the number of moles of hydrogen must be divided into two parts: one part $n_{H_{2,A,\;0}}$ must be capable of full interaction with anthracene, and the second part $n_{H_{2,bal}}$ turns out to be ballast and has the form:

(46) $$n_{H_{2,\;0}}=n_{H_{2,A,\;0}}+n_{H_{2,\;bal}}$$

In turn, the anthracene part of hydrogen in the reaction process will be consumed in proportion to the fractional conversion of anthracene *α*.

The remaining part of anthracene hydrogen is calculated as follows:

(47) $$n_{H_{2,\;A}}=n_{A,0}(1-\alpha )$$

The balance expression for the molar hydrogen content at any moment τ corresponding to the degree of converted *α* is calculated as follows:

(48) $$n_{H_{2,\;\propto }}=n_{A,0}\left(1-\alpha \right)+n_{H_{2,\;bal}}=n_{A,0}-n_{A,0}\alpha +n_{H_{2,\;bal}}=n_{H_{2},0}-n_{A,0}\alpha$$

From the molar content of hydrogen, we turn to expressing it through pressure. To do this, we use the Clapeyron–Mendeleev equation:

*PV* = *nRT* (49)

where *n* is the number of moles of a gaseous substance.

From (52), we obtain:

(50) $$n=\frac{PV}{RT}$$

During each series of experiments, the temperature is set constant, and the volume of hydrogen above the mixture of anthracene and dihydroanthracene remains constant.

Therefore, a direct proportion between *n* and *P* is maintained (hydrogen is consumed in proportion to the decrease in pressure):

(51) $$n=P\frac{V}{RT}$$

Substituting (53) into (51), we obtain:

(52) $$P_{\alpha }=\frac{V}{RT}=n_{H_{2},0}-n_{A,0}\alpha$$

Instead of $n_{H_{2},0}$, we substitute its expression in terms of pressure:

(53) $$n_{H_{2}0}=P_{0}\frac{V}{RT}$$

where *P*_0_ is the initial pressure of hydrogen at temperature *T*:

(54) $$P_{\alpha }\frac{V}{RT}=P_{0}\frac{V}{RT}-n_{A,0}\alpha$$

Or:

(55) $$P_{\alpha }=P_{0}-\alpha \frac{n_{A,0}RT}{V}$$

At equilibrium *α = α_eq_*, expression (58) will take the form:

(56) $$P_{\alpha_{eq}}=P_{0}-\alpha_{eq}\frac{n_{A,0}RT}{V}$$

In the experiment, the initial number of moles of hydrogen was set constant, because hydrogen was pumped at a pressure of *P* = 30 atm = 30/10.2 = 2.94 MPa = 2.94⋅10^6^ Pa at *T* = 293 K and *V* = 50⋅10^−6^ m^3^. Therefore, we find $n_{H_{2,0}}$—the number of moles of hydrogen:

$$n_{H_{2,0}}=\frac{P_{0}V}{RT_{0}}=\frac{2.94\cdot 10^{6}\cdot 50\cdot 10^{-6}}{8.31441\cdot 293}=6.034\cdot 10^{-2}\mathrm{mol}$$

When heated to the experimental temperature *T*, the number of moles does not change, as does the volume occupied by hydrogen, so the initial pressure at temperature *T* will be determined by the expression:

(57) $$\frac{P_{T}V}{RT}=\frac{P_{0}V}{R\cdot T_{0}}=n_{H_{2},0}=n_{H,\delta }+n_{H_{2},A_{0}}$$

Due to the constancy of *T*, the initial pressure of *RT* can be divided into two partial parts—ballast *P_bal_* and anthracene–related *P_bal_*:

*P_T_* = *P_bal,T_* + *P_A,T_* (58)

In turn, the anthracene part decreases in proportion to the age of *α*, i.e.,

*P_A,τ_* = *P_A,T_* (1 − *α*) (59)

From where the total pressure will be equal for any point in time:

*P_T,τ_* = *P_bal,T_* + *P_A,T_* (1 − *α*) (60)

Since the ballast part of the initial pressure is equal to *P_bal_,_T_ = P_T_* − *P_A,T_*, we obtain:

(61) $$P_{T,\tau }=P_{T}-P_{A,T}+P_{A,T}\left(1-\alpha \right)=P_{T}-P_{A,T}+P_{A,T}-aP_{A,T}$$

(62) $$P_{T,\tau }=P_{T}-\alpha P_{A,T}$$

$P_{T}=\frac{n_{H_{2},0}\cdot RT}{V}$, accordingly $P_{A,T}=\frac{n_{A,0}\cdot RT}{V}$, Therefore, the current pressure is:

(63) $$P_{T,\tau }=\frac{n_{H_{2,}0}\cdot RT}{V}-\frac{n_{A,0}RT}{V}\alpha$$

The found expression for *P_a_* and $P_{\alpha_{eq}}$ is substituted into the original expression (48)

(64) $$\left(P_{0}-\alpha \frac{n_{A,0}RT}{V}-P_{0}+\alpha_{eq}\frac{n_{A,O}RT}{V}\right)=\frac{D}{\delta }\cdot \frac{n_{A,O}RT}{V}F\left(\alpha_{eq}-\alpha \right)$$

The calculation equation in differential form is as follows:

(65) $$\frac{\mathrm{d}\alpha }{\mathrm{d}\tau }=\frac{D}{\delta }\cdot \frac{n_{A,0}RT}{V}F\left(\begin{array}{cc} \alpha_{eq} & -\alpha \end{array}\right)$$

For each isotherm $\frac{D}{\delta }\cdot \frac{n_{A,0}RT}{V}\cdot F=const_{\left(T\right)}=\alpha_{T}$; therefore, for further transformations, we fix a simplified notation:

(66) $$\frac{\mathrm{d}\alpha }{\mathrm{d}\tau }=\alpha_{T}\left(\alpha_{eq}-\alpha \right)$$

Separation of variables:

(67) $$\frac{\mathrm{d}\alpha }{\alpha_{eq}-\alpha }=\alpha_{T}\mathrm{d}\tau$$

After integration, we obtain:

(68) $$\int_{0}^{\alpha }\frac{\mathrm{d}\alpha }{\alpha_{eq}-\alpha }={-\left.\ln\;\left(\alpha_{eq}-\alpha \right)\right|}_{0}^{a}=-\ln\;\left(\alpha_{eq}-\alpha \right)+\ln\;\left(\alpha_{eq}-0\right)=\ln\;\alpha_{eq}-\ln\;\left(\alpha_{eq}-\alpha \right)=\;=\ln\;\frac{\alpha_{eq}}{\alpha_{eq}-\alpha }$$

(69) $$\int_{0}^{\alpha }\alpha_{T}\mathrm{d}\tau =\alpha_{T}\tau$$

The calculation formula in integral form:

(70) $$\ln\;\frac{\alpha_{eq}}{\alpha_{eq}-a}=\alpha_{T}\tau$$

When *α* = 0 $\ln\;\frac{\alpha_{eq}}{\alpha_{eq}-a}=ln1=0$ means τ = 0.

When *α* = *α_eq_* $\ln\;\frac{\alpha_{eq}}{\alpha_{eq}-\alpha_{eq}}=\ln\;\frac{\alpha_{eq}}{0}=\ln\;\infty =\infty$ means τ = ∞.

This corresponds to the physical meaning of the connection α and τ.

In Formula (73), there are two unknown quantities *α_eq_* and *α_T_*. They can be found by one of the EKA methods. The simplest method is to solve two equations with two unknowns, with a search of all non-repeating pairs of experimental data (*α_i_*, *τ_i_*) and (*α_j_*, *τ_j_*), followed by averaging the results.

For the *i*-th experiment, Formula (73) will take the form:

(71) $$\ln\;\frac{\alpha_{eq}}{\alpha_{eq}-\alpha_{i}}=\alpha_{T}\tau_{i}$$

For the *j*-th experiment:

(72) $$\ln\;\frac{\alpha_{eq}}{\alpha_{eq}-\alpha_{j}}=\alpha_{T}\tau_{j}$$

By dividing Equation (74) by (75), we obtain:

(73) ln aeqαeq−αiln αeqαeq−aj=τiτj

At the same time, *α_T_* is reduced and Equation (76) turns out to have only one unknown, so this unknown *α_eq_* can be found numerically (*α_eq_* is not released from under the sign of the logarithm here).

The numerical solution procedure consists in such a selection of *α_eq_*, in which the left side of Equation (76) will be equal to the known right. In this case, you cannot set *α_eq_ ≤ α_j_* if *τ_i_ < τ_j_*. This also guarantees the condition *α_eq_ > α_j_*, since *α_j_ ≤ α_i_*. The initial data and the results of calculating the equilibrium fractional conversion of anthracene *α_eq_* (starting from 60 min) are shown in Table 2

Calculation of the coefficient *α_T_*. Equation (73) is converted to the form:

(74) $$\alpha_{T}=\frac{1}{\tau }\ln\;\frac{\alpha_{eq}}{\alpha_{eq}-\alpha },{мин}^{-1}$$

For each isotherm, it is necessary to calculate *α_T_* at all points: substituting in (76) the corresponding values *α*, *τ,* and a constant value *α_eq_* for a given temperature. Then, carry out the averaging of the *α_T_*.

Knowing the *α_T_* and *α_eq_* for each isotherm, it is possible to calculate the dependence of α on τ by (73) and compare these data with the experimental results.

### 3.7. Determination of Activation Energy by Diffusion Coefficients

The designation of the coefficient is as follows:

(75) $$\alpha_{T}=\frac{D}{\delta }\cdot \frac{n_{A,0}RT}{V}\cdot F=const_{\left(T\right)}$$

In this expression, the unknown value is *D⁄δ*—the diffusion coefficient divided by the thickness of the diffusion layer. Although *δ* is usually a constant value, its specific value is unknown, so it is impossible to find *D* in its pure form through *α*. However, it will be possible to find the activation energy through the dependence *α_T_ = f(T)* if it is converted to the form of the Arrhenius equation.

To do this, first, you need to represent, as usual, the diffusion coefficient in the form *D = D*_0_ − *exp(Ed / RT)*, where *D*_0_ is the frequency factor, *Ed* is the diffusion activation energy. We substitute this expression into the formula for *α_T_*:

(76) $$\alpha_{T}=\frac{n_{A0}\cdot R\cdot T\cdot F\cdot D_{0}}{\delta \cdot v}\exp\;\left(-\frac{E_{d}}{RT}\right)$$

Secondly, the pre-exponential fraction must be freed from temperature in order to ensure the constancy of the pre-exponential part at any temperature:

(77) $$a^{\prime }=\frac{\alpha_{T}}{T}=\frac{n_{A,0}\cdot R\cdot F\cdot D_{0}}{\delta \cdot V}\exp\;\left(-\frac{E_{d}}{RT}\right)$$

Then, you should enter the designation of a new constant for any temperature:

(78) $$b=\frac{n_{A,0}\cdot R\cdot F\cdot D_{0}}{\delta \cdot V}$$

we obtain a simplified expression for (80):

(79) $$a^{\prime }=b\exp\;\left(-\frac{E_{d}}{RT}\right)$$

After the logarithm, we obtain the equation:

(80) $$\ln\;a^{\prime }=\ln\;b-\frac{E_{d}}{R}\cdot \frac{1}{T}$$

from which, in the coordinates $\ln\;a^{\prime }-\frac{1}{T}$, we find the angular coefficient $C=-\frac{E_{d}}{R}$ and then *E_d_ = −R*, as well as the value of d=lnb, from where $b=e^{d}$.

After determining *b* from (81), we find the value:

(81) $$\frac{D_{0}}{\delta }=\frac{b\cdot V}{n_{A,0}\cdot R\cdot F\cdot D_{0}}$$

substituting all known values into the right part.

As a result, we find the final expression for the reduced diffusion coefficient:

(82) $$D\prime =\frac{D_{0}}{\delta }\exp\;\left(-\frac{E_{d}}{RT}\right)$$

The initial data for calculating the activation energy and diffusion coefficient using the least squares method are presented in Table 9.

The calculation of values is as follows:

(83) $$C=\frac{-E_{d}}{R}$$

$$E_{d}=2670.6\cdot R=2670.6\cdot 8.314\approx 22.2\;\mathrm{kJ/mol}$$

(84) $$F=\frac{3m_{0}}{\tau \cdot p}=\frac{3\cdot 0.00015}{0.00005\cdot 5.03}=1.8$$

(85) $$\frac{D_{0}}{\delta }=\frac{b\cdot v}{n_{A0}\cdot R\cdot F}=\frac{1.53\cdot 10^{-11}\cdot 5\cdot 10^{-5}}{1.683\cdot 10^{-2}\cdot 8.314\cdot 1.8}={2.59\cdot 10}^{-6}$$

*b = e*
^−4.37^ (86)

Judging by the magnitude of the activation energy, the process of anthracene hydrogenation proceeds mainly in the diffusion region.

## 4. Conclusions

As a result of the conducted studies of the process of anthracene hydrogenation in the presence of a mixture of pyrite and aluminum oxide at various temperatures, it was found that an increase in temperature (from 648 K to 698 K) leads to an increase in the rate of anthracene conversion. The use of equilibrium kinetic analysis makes it possible to establish the relationship between direct and reverse reactions for a better understanding of the catalytic properties of a mixture of aluminum oxide and pyrite in the process of anthracene hydrogenation, as well as to establish the dependence of the reaction rate on time and temperature. The effect of diffusion and mass transfer on the kinetics of the process becomes more noticeable with an increase in temperature, when the reagent molecules do not have time to reach the active centers of the catalyst in time due to the porosity of the catalyst, its deactivation, competing adsorption processes, etc.

It was found that the process of anthracene hydrogenation in the presence of a mixture of FeS_2_ and Al_2_O_3_ is limited from the kinetic to the diffusion region as the temperature increases, which is confirmed by the values of the activation energy of the direct (39.4 kJ/mol) and the reverse reaction (13.04 kJ/mol). An increase in the rate constants of the direct anthracene hydrogenation reaction with an increase in temperature is significantly associated with an increase in the number of effective collisions of reagent molecules at the active centers of the catalyst, which reduces the likelihood of a dehydrogenation reaction. In addition, it was found that with increasing temperature, the Gibbs energy decreases, which makes the process of forward reaction (hydrogenation) more thermodynamically advantageous.

The values of enthalpy and entropy of the reactions calculated using the Gibbs–Helmholtz equation indicate the endothermic nature of the process. The positive value of entropy also suggests an increase in disorder in the system, which may be due to a partial loss of aromaticity of anthracene during hydrogenation.

## Figures and Tables

**Table 1:** The values of the fractional conversion (α) of anthracene obtained during the hydrogenation of anthracene in the presence of a mixture of pyrite and aluminum oxide at different temperatures.

Duration *τ,* min	648, K *α*	673, K *α*	698, K *α*
45	0.259	0.350	0.442
60	0.350	0.441	0.540
75	0.47	0.56	0.65
90	0.50	0.58	0.68

**Table 2:** Determination of the equilibrium fractional conversion of reaction at different temperatures.

$\frac{\tau_{i}}{\tau_{j}}$	T = 648 K		T = 673 K		T = 698 K	
	$\frac{\alpha_{i}}{\alpha_{j}}$	*α_eq_*	$\frac{\alpha_{i}}{\alpha_{j}}$	*α_eq_*	$\frac{\alpha_{i}}{\alpha_{j}}$	*α_eq_*
$\frac{60}{75}$	$\frac{0.40}{0.41}$	0.419	$\frac{0.49}{0.51}$	0.534	$\frac{0.59}{0.62}$	0.663
$\frac{60}{90}$	$\frac{0.40}{0.42}$	0.427	$\frac{0.49}{0.53}$	0.551	$\frac{0.59}{0.64}$	0.668
$\frac{75}{90}$	$\frac{0.41}{0.42}$	0.433	$\frac{0.51}{0.53}$	0.565	$\frac{0.62}{0.64}$	0.671
		$\bar{\alpha_{eq}}=0.426$		$\bar{\alpha_{eq}}=0.550$		$\bar{\alpha_{eq}}=0.679$

**Table 3:** Equilibrium constants of anthracene hydrogenation at different temperatures.

*T*, K	648	673	698
*K_eq,T_,* Pa^−1^	1.295·10^−1^	2.138·10^−1^	3.726·10^−1^

**Table 4:** Equilibrium constants and Gibbs energies of the anthracene hydrogenation reaction.

*T*, K	648	673	698
*K_eq_*, atm^−1^	1.46·10^−2^	2.487·10^−2^	3.63·10^−2^
${∆G}_{T}^{0}$, J/mol	2277.28	2067.01	1924.29

**Table 5:** Calculation of the data of the feedback rate constant.

*T*, K	648	673	698
*K_2_*, s^−2^	1.717·10^−4^	1.875·10^−4^	1.61·10^−4^
*ln K*_2_	−8.47	−8.582	−8.734

**Table 6:** Constants of the rate and energy of activation of the forward and reverse reactions.

*T*, K	*α_eq_*	Forward Reaction		Reverse Reaction	
		*K*_1_, s^−1^	*E*, kJ/mol	*K*_2_, s^−1^	*E*, kJ/mol
648	0.426	2.506·10^−6^		1.717·10^−4^	
673	0.550	4.663·10^−6^	39.4	1.875·10^−4^	13.04
698	0.679	5.844·10^−6^		1.61·10^−4^	

**Table 7:** Calculation of the equilibrium fractional conversion of anthracene.

*T* = 648 K			*T* = 673 K			*T* = 698 K		
*τ_i_*/*τ_j_*	*α_i_*/*α_j_*	*α_eq_*	*τ_i_*/*τ_j_*	*α_i_*/*α_j_*	*α_eq_*	*τ_i_*/*τ_j_*	*α_i_*/*α_j_*	*α_eq_*
0.80	0.40/0.41	0.41	0.80	0.48/0.51	0.53	0.80	0.59/0.62	0.65
0.66	0.40/0.42	0.42	0.66	0.49/0.53	0.56	0.66	0.59/0.64	0.66
0.83	0.41/0.42	0.43	0.83	0.51/0.53	0.56	0.83	0.62/0.64	0.66
		$\bar{\alpha_{eq}}=0.42$			$\bar{\alpha_{eq}}=0.54$			$\bar{\alpha_{eq}}=0.66$
						0.57	0.59/0.66	0.67
						0.71	0.62/0.66	0.68
						0.85	0.64/0.66	0.69
								$\bar{\alpha_{eq}}=0.67$

**Table 8:** Calculation of the fractional conversion of anthracene *α_τ_* depending on time.

*T* = 648 K			*T* = 673 K			*T* = 698 K		
*α_eq_* = 0.45			*α_eq_* = 0.58			*α_eq_* = 0.67		
*τ*, мин	*α*	*α*·10^−2^	*τ*, мин	*α*	*α*·10^−2^	*τ*, мин	*α*	*α*·10^−2^
15	0.143	2.55	15	0.219	3.15	15	0.30	3.95
30	0.161	1.47	30	0.277	2.15	30	0.364	2.604
45	0.259	1.90	45	0.350	2.04	45	0.442	2.39
60	0.350	2.49	60	0.441	2.36	60	0.537	2.69
75	0.401	2.94	75	0.557	4.22	75	0.68	4.61
90	0.42	2.95	90	0.58	6.69	90	0.66	4.55
		$\bar{a_{648}}$= 1.43⋅10^−2^			$\bar{a_{673}}$= 2.06⋅10^−2^			$\bar{a_{698}}$= 2.08⋅10^−2^

**Table 9:** Initial data for the calculation of the activation energy and diffusion coefficient using the least squares method.

*T*, K	1*/T*	lna *’*	$\frac{\ln\;a^{’}}{T}$	$\frac{1}{T^{2}}$
648	1.54·10^−3^	−8.415	−1.299·10^−2^	2.381·10^−6^
673	1.48·10^−3^	−8.352	−1.24·10^−2^	2.308·10^−6^
698	1.43·10^−3^	−8.118	−1.163·10^−2^	2.052·10^−6^
	4.45·10^−3^	−24.885	−3.702·10^−2^	6.641·10^−6^

## Data Availability

Data are contained within the article.
